# Editorial: Small and mighty: Brain capillaries in health and disease

**DOI:** 10.3389/fnmol.2022.1108978

**Published:** 2022-12-13

**Authors:** Oliver Bracko, Osama F. Harraz

**Affiliations:** ^1^Department of Biology, University of Miami, Coral Gables, FL, United States; ^2^Department of Neurology, University of Miami-Miller School of Medicine, Miami, FL, United States; ^3^Department of Pharmacology, Larner College of Medicine, and Vermont Center for Cardiovascular and Brain Health, University of Vermont, Burlington, VT, United States

**Keywords:** capillaries, cerebral blood flow, capillary stalling, neurovascular coupling, pericytes

## Introduction

Capillaries are the smallest blood vessels and the major site for oxygen exchange, glucose delivery, and waste removal. Within the brain, the “*small and mighty*” capillaries have become an area for active research in the past few years. Recent studies have shown that brain capillaries are key sensors of chemical cues derived from surrounding neurons and astrocytes, leading to vascular responses. Capillaries also sense intravascular forces, which can change during capillary occlusions that are commonly observed in disease (Longden et al., 2017, 2021; Harraz et al., 2018a,b, 2022; Cruz Hernández et al., 2019; Bracko et al., 2021; Sancho et al., 2022). The capillary wall is formed of capillary endothelial cells and surrounding pericytes. Both cell types have been implicated in capillary function and dysfunction (Sagare et al., 2013; Kisler et al., 2017; Nortley et al., 2019; Dabertrand et al., 2021; Mughal et al., 2021). Other cell types—such as astrocytes, blood cells, and microglia—interact with capillaries leading to changes in cerebral blood flow. The Frontiers Research Topic [*Small and mighty: Brain capillaries in health and disease*] covers some aspects of capillary involvement in neurovascular coupling, capillary stalling, and pericyte pathophysiology.

## Frontiers Research Topic

Cerebral blood flow (CBF) changes can attribute to brain capillary occlusion. Stalled capillaries within a capillary network block capillary blood flow from seconds to minutes and can cause local changes in oxygen levels and metabolites (Reeson et al., 2018; Bracko et al., 2021). In fact, neuronal networks rely on local neurovascular coupling which regulates local capillary blood flow to fulfill their metabolic demand. In the present Research Topic, the study by Reeson et al. used *in vivo* imaging and found that an enriched environment and exercise reduced the number of capillary stalls in the cortex of mice. Furthermore, this study shows that neuronal activity itself reduced the number of obstructions. Overall, their data indicate that capillary obstruction might be bidirectionally regulated. This study therefore sheds new light on the various causes contributing to capillary stalls that are observed in neurodegenerative and cardiovascular diseases.

Mild traumatic brain injury (mTBI) significantly impacts the microvasculature and leads to blood flow reductions in capillary networks (Witkowski et al., 2019; Han et al., 2020). The article by Wu et al. developed a modified dielectric elastomer actuator inducing an mTBI by mechanical stretching. The model was confirmed using transcriptomics of human-derived pericytes, and the results indicated an increase in mTBI-induced genes after injury. This novel model of stretch-induced mTBI could be a powerful tool to further investigate the contribution of pericytes to capillary function.

The role of pericytes in neurovascular coupling in health and disease has been an active area for research. Different approaches have been used to study pericyte physiology and pathophysiology. In the current Frontier Research Topic, Nielson et al. provide a succinct and detailed non-genetic approach to ablate pericytes. Such an approach employs targeted infrared laser light to induce pericyte death, and therefore helps circumvent the unwanted, often complex outcomes of genetic pericytes ablation. Major advantages include minimal disruptions of the blood brain barrier and the no need for exogenous photosensitizers. This Methods article therefore provides valuable insights that will help guide research efforts examining the consequences of pericyte loss on CBF.

Stroke patients suffer from impaired neurovascular coupling as well as CBF deficits. These impairments accelerate neurological deficits and are not limited to the stroke infarct. Li et al. explored the mechanisms underlying the peri-infarct impairments. Using an established model of stroke in rats, the authors showed that capillary dilation is impaired in non-infarct cortical tissues, despite normal neural activity and vascular contractility. Through a series of clever experiments, Mishra and colleagues identified an increase in the levels of the vasoconstrictor 20-hydroxyeicosatetraenoic acid (20-HETE) in the cortex to be involved in neurovascular impairment beyond the infarct. Not only does this study explain how non-infarct areas are impacted, but also it introduces a candidate therapeutic intervention to restore blood flow after stroke.

## Closing remarks

While the interest in capillaries in the brain has gained momentum recently, many more open questions in this emerging field await investigations. We believe that understanding capillary physiology and pathophysiology has the potential to pave the path to therapeutic interventions that are much needed for cardiovascular and neurodegenerative diseases.

## Author contributions

All authors listed have made a substantial, direct, and intellectual contribution to the work and approved it for publication.
